# Paroxysmal Nocturnal Hemoglobinuria: An Underestimated Cause of Pediatric Thromboembolism

**DOI:** 10.1055/s-0040-1702155

**Published:** 2020-02-20

**Authors:** Christina Griesser, Michael Myskiw, Werner Streif

**Affiliations:** 1 Department of Paediatrics I, Medical University of Innsbruck, Innsbruck, Austria; 2 Institute for Diagnostic and Interventional Radiology, RoMed Hospital Rosenheim, Rosenheim, Germany

**Keywords:** child, hemolysis, PIG-A gene, paroxysmal nocturnal hemoglobinuria, thrombosis

## Abstract

Paroxysmal nocturnal hemoglobinuria (PNH) is a chronic disease caused by complement-mediated hemolysis. Clinical symptoms include intravascular hemolysis, nocturnal hemoglobinuria, thromboses, cytopenia, fatigue, abdominal pain, and a strong tendency toward bone marrow failure. It is a rare disease, especially in children, with high mortality rates without appropriate treatment.

We here present the case of a 17-year-old girl with unprovoked muscle vein thrombosis. Flow cytometric analysis showed deficiency of glycosyl-phosphatidylinositol-anchored membrane proteins on all three hematopoietic cell lines and confirmed the diagnosis of PNH. Treatment with the monoclonal antibody eculizumab achieved long-term remission.

As flow cytometry is normally not part of the routine diagnostics for pediatric thrombosis, awareness is crucial and PNH is important to consider in all children with thrombosis at atypical sites and abnormalities in blood counts with regard to hemolysis and cytopenia.

## Introduction

Thromboses in childhood are rare conditions with incidence rates of ∼30 per 10,000 hospital admissions. 1 The incidence follows a bimodal pattern with a peak occurring in newborns and adolescents. In contrast to adults, idiopathic thromboses are rare and most are associated with predisposing risk factors. Catheters are the most common cause, with others being trauma, drugs, and acute and chronic diseases. In young women, hormonal contraception plays a significant role. 1 Paroxysmal nocturnal hemoglobinuria (PNH) is an extremely rare cause of thrombosis in childhood.

PNH is an acquired clonal disorder of the hematopoietic stem cells caused by a somatic mutation of the phosphatidylinositol glycan class A (PIG-A) gene. 2 3 This leads to an early defect in the biosynthesis of the glycosyl-phosphatidylinositol (GPI) anchor. Consequently, GPI-anchored membrane proteins as decay accelerating factor (DAF, CD55) and membrane inhibitor of reactive hemolysis (MIRL, CD59) are lacking. 4 5 6 These are crucial for protecting cells against spontaneous actions of the complement system. 5 6 7 The mutation in the PIG-A gene may have an effect on one or on all three hematopoietic cell lines and cause pancytopenia. 3 The unusual susceptibility of the cell membrane causes intravascular hemolysis. Hemolysis and platelet activation lead to systemic complications—anemia and fatigue, life-threatening thromboembolic events, chronic kidney disease, pulmonary hypertension, and cardiovascular and neurological impairments. 7 8 9 Pathogenesis of platelet activation in PNH are complement-mediated activation, nitric oxide deficiency, direct effects of free hemoglobin, increased concentration of reactive oxygen species, thrombin activation, and endothelial dysfunction. 9 Without adequate treatment, up to 35% of patients with PNH die within 5 years of diagnosis. 3 Of all PNH deaths, 40 to 67% occur due to venous or arterial thrombosis. 3 Thromboses are often located at atypical sites including sinovenous, portal, and abdominal veins. 3 So far, the only curative therapy option is allogeneic stem cell transplantation. Another treatment option is the humanized monoclonal antibody eculizumab. It inhibits the terminal complement factor and prevents formation of the membrane attack complex. This significantly reduces hemolysis and the formation of thrombosis. After 36 months of treatment, the reported overall survival rate with eculizumab was 97.6%. 10 11 In 2014, Reiss et al published a prospective multicenter study that encourages the use of eculizumab in children. 12

## Case Presentation

A 17-year-old girl presented with pain in the right lower leg for 3 days. Until then she had always been healthy. For about 2 months she had been taking a combined oral contraceptive with antiandrogenic effect and well-known high risk for thrombosis. Possible causes of pain included muscle distension, myositis, deep vein thrombosis, and muscle vein thrombosis. Last-mentioned was confirmed in the right lower leg by ultrasound. Blood tests revealed an elevated D-Dimer level of 6.5 mg/L, anemia (hemoglobin 5.341 mmol/L), pancytopenia, and an elevated lactate dehydrogenase level of 1,314 U/L. Hematopoietic examination by bone marrow aspiration followed. Flow cytometry showed a CD55, CD59, and fluorescein-labeled proaerolysin (FLAER) deficiency on granulocytes, monocytes, and erythrocytes, which confirmed the suspected diagnosis of PNH. In addition to anticoagulation with phenprocoumon, a vitamin K antagonist, periodic intravenous administration of eculizumab was started and the patient has been in remission for more than 2 years now.

## Single-Center Experience with Pediatric Thromboembolism

**Fig. 1:**
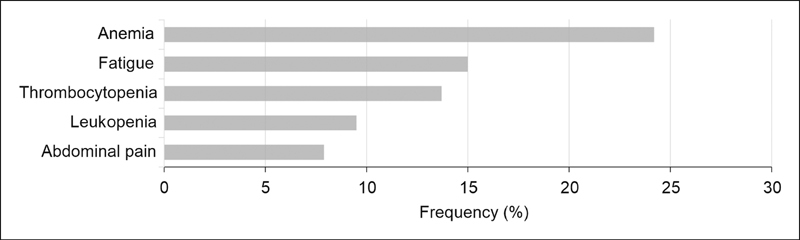
Symptoms and signs typical for paroxysmal nocturnal hemoglobinuria in a cohort of 150 children with confirmed thrombosis treated at the Department of Paediatrics at the Medical University of Innsbruck, Austria, between 2004 and 2013.

We performed a retrospective single-center study to investigate the prevalence of symptoms and signs typical for PNH in children presenting with thrombosis. The cohort consisted of 150 children under the age of 18 years who suffered from thrombotic or thromboembolic events and were treated at the Department of Paediatrics of the Medical University of Innsbruck, Austria, between 2004 and 2013. The data were analyzed while applying the diagnostic criteria for PNH: thrombosis, anemia, leukopenia, thrombocytopenia, abdominal pain, and fatigue ( Fig. 1 ). Twenty-five (16.7%) of the children corresponded to at least four criteria (including thrombosis), but none of these children was finally diagnosed with PNH.

## Discussion

PNH is a rare disease with onset usually in adulthood, but it also occurs in children. The reported incidence rate is 1.3 cases per million inhabitants per year. The prevalence is unknown, but it is assumed that the disease is under- and often misdiagnosed. On average there is a delay in diagnosis of more than 2 years. Explanations therefore are, on the one hand, the rarity of the disease, its nonspecific symptoms, and the clinical association with other bone marrow failure diseases, but, on the other hand, also the lacking awareness among physicians. 2 Moreover, there are strong distinctions between the presentation of adult and pediatric patients with PNH. In contrast to adults, where hemoglobinuria is found as presenting symptom in 50% of cases, only ∼10% of pediatric patients with PNH present with it. 2 4 13 In children, the frequency of coexisting bone marrow failure syndromes is ∼80%. These may present simultaneously or one may evolve into another and can lead to misdiagnosis. 2 13 There is a strong clinical and pathophysiological correlation between PNH and aplastic anemia, myelodysplastic syndrome, and acute myeloid leukemia. Thus, these diseases can be considered predisposing factors for the development of PNH. In contrast, in adults the frequency is ∼30%. 2 3 14

Historically, the delay between presentation and diagnosis has been attributed to the lack of sensitivity of the acid Ham test and the sucrose lysis test. 10 13 Today, the method of choice for confirming PNH is flow cytometry. It analyzes the absence of GPI-anchored surface proteins and detects PNH clones. 13 Distinction is made between type I cells with normal expression of surface proteins, type II cells with partial expression, and type III cells with complete deficiency. 15 A new approach to the diagnosis of PNH is to use FLAER as it binds selectively to the GPI anchor without causing cell lysis. 4 Normally, flow cytometry is not part of the routine diagnostics for thrombosis. It requires about 1 mL of peripheral blood from EDTA tubes and therefore it is also suitable for children of all ages. 8

Diagnostic criteria for PNH are thrombosis, anemia, leukopenia, thrombocytopenia, abdominal pain, and fatigue. The frequency of thromboses in children with verified PNH varies in literature between 20 and 50%. 2 10 13 Asian children with PNH appear to be less at risk. 16 Anemia is often associated with symptoms including weakness, paleness, shortness of breath, and fatigue. In our cohort of 150 children presenting with thrombosis, 24.5% of the children had an anemia and 15% indicated weakness or fatigue ( Fig. 1 ).

The frequency of leukopenia was 9.5% ( Fig. 1 ). Thrombocytopenia occurred in 13.7% of the children ( Fig. 1 ), in some cases resulting in bleeding, epistaxis, or subconjunctival hemorrhages. In accordance with published literature on children with PNH, in our cohort abdominal pain occurred also with a frequency of 7.9% ( Fig. 1 ). 2

From this cohort of 150 children, 25 (16.7%) suffered from at least four symptoms/signs typical for PNH. In Table 1 , we present a summary of the presenting laboratory characteristics of these 25 children, who all presented with thrombosis and another three criteria suspicious for PNH. Overall, 84% of the children had decreased hemoglobin levels (5.4 ± 0.7 mmol/L), 64% showed a decreased platelet, and 52% a lower leukocyte count ( Table 1 ). As typical for hemolysis, 52% had elevated reticulocyte numbers, 36% elevated bilirubin, and 20% elevated lactate dehydrogenase (LDH) plasma levels ( Table 1 ). However, none of these children was diagnosed with PNH. Instead, 5 (20%) of the 25 children, corresponding to at least four symptoms/signs typical for PNH, suffered from leukemia as examined by bone marrow puncture and were in turn treated with corticosteroids and asparaginase. This explains the unusual finding that thrombosis occurred with relevant bleedings, as mentioned above. Six (24%) children were diagnosed with other malignancies, and another six (24%) with cardiomyopathies.

**Table 1:** Presenting laboratory characteristics of 25 children with confirmed thrombosis at the Department of Paediatrics at the Medical University of Innsbruck, Austria, between 2004 and 2013

Presenting symptom/sign	Children with thrombosis and symptom/sign suspicious for PNH a	Mean value ± standard deviation	Normal range	Unit
Decreased hemoglobin	21 (84%)	5.4 ± 0.7	6.3–7.9	mmol/L
Decreased platelets	16 (64%)	115,000 ± 54,000	206,000–459,000	cells/µL
Decreased leukocytes	13 (52%)	3,200 ± 1,500	6,000–13,500	cells/µL
Elevated reticulocytes	13 (52%)	31 ± 7	9.9–18.2	‰
Elevated bilirubin	9 (36%)	51.3 ± 49.6	3.4–17.1	µmol/L
Elevated LDH	5 (20%)	2,210 ± 1,530	225–600	U/L

Abbreviations: LDH, lactate dehydrogenase; PNH, paroxysmal nocturnal hemoglobinuria.

Note: All patients were suspicious (>3 criteria for PNH) for, but not diagnosed with PNH. The normal reference range and the according units are indicated.

a The total number ( *n* ) of patients (out of 25) and proportion (%) are listed for each parameter, given as mean value plus standard deviation.

As this is a retrospective data analysis, not all children were examined by bone marrow puncture and/or flow cytometry. However, PNH as cause of thrombosis could be ruled out in all 25 children with signs/symptoms of PNH and thrombosis, since follow-up data and personal contacts with caregivers, respectively, were available and even more part of the presented study.

As already mentioned, the prevalence of PNH is low and thus it is not surprising that since 2004 and after 2013 only one child has been diagnosed with PNH at the Paediatric Department of the Medical University of Innsbruck, Austria. However, PNH is a progressive disease and a prompt and accurate diagnosis is important for the health of the patient. In accordance with our findings, Patriquin et al recently published a review article proposing that the diagnosis of PNH should be considered in five clinical scenarios, abbreviated by the acronym CATCH: cytopenia, aplastic anemia/myelodysplasia, thrombosis, Coombs'-negative hemolysis, and hemoglobinuria. 17 Both, our case presentation and retrospective single-center study show that in all children with thrombosis, intravascular hemolysis, and cytopenia, it is worth considering PNH as underlying disease to prevent delayed diagnosis and to initiate the appropriate treatment including eculizumab.
